# Biocontrol Efficacy and Mechanisms of *Bacillus velezensis* AP6 Against Brown Rot in Yam by *Fusarium solani*

**DOI:** 10.3390/jof12050345

**Published:** 2026-05-07

**Authors:** Yangtian Li, Bowen Tang, Jinchi Xie, Yaohui Pan, Taixin Yang, Xiaoxiao Feng, Bin Zhao, Yingchao Liu

**Affiliations:** 1 College of Plant Protection, Hebei Agricultural University, Baoding 071001, China; 2 College of Agronomy, Hebei Agricultural University, Baoding 071001, China

**Keywords:** *Bacillus velezensis*, *Fusarium solani*, yam brown rot, biocontrol mechanism, field control efficacy

## Abstract

Yam is a valuable traditional Chinese medicinal and nutritional crop that has gained significant popularity in recent years. However, the production of yam is severely impacted by brown rot caused by *Fusarium solani*, which affects both yield and quality. In this study, we isolated a promising biocontrol strain, designated AP6, from the rhizospheric soil of healthy yam. Strain AP6 exhibited strong antagonistic activity against *F. solani*, with an inhibition rate of 60.2%, and demonstrated broad-spectrum antagonistic activity. Based on morphological, physiological, biochemical characteristics, and whole-genome sequencing, this strain was identified as *Bacillus velezensis*. Strain AP6 can produce siderophores, amylase, protease, cellulase, and form biofilms. It can also change the hyphal morphology of *F. solani*, inhibit spore germination, reduce the viability of pathogens, and alleviate the disease severity of yam. Additionally, strain AP6 was shown to induce the accumulation of reactive oxygen species in yam, thereby enhancing the yam’s defense responses. Field application trials demonstrated that a wettable powder formulation of strain AP6, when combined with commercial metalaxyl-M and fludioxonil, significantly reduced the incidence of brown rot in yam. These findings indicate that *B.velezensis* AP6 is a promising biocontrol agent, providing a practical and sustainable approach for integrated disease management in yam.

## 1. Introduction

Soil-borne diseases, caused by pathogens residing in soil and decaying crop residues, pose a significant threat to crop health and productivity worldwide [1]. These diseases are often characterized by complex interactions among multiple pathogens, complicating management and control strategies [2]. Notably, soil-borne pathogens such as *F. solani*, *Fusarium oxysporum*, and *Rhizoctonia solani* can persist in soil for extended periods, continually threatening a wide range of crops, including wheat, tomato, and ginseng [3,4,5,6].

In China, yam (*Dioscorea opposita* Thunb.), a perennial climbing herb, occupies a significant position in both culinary and medicinal contexts. It is valued for its dual role as a food source serving as both a vegetable and a staple and as an important component in traditional Chinese medicine [7]. The main production areas of yam in China include Hebei, Henan, Zhejiang, and other provinces [8]. The increase in social consumer demand and the enhancement of economic benefits have, as a result, led to a continuous expansion in the scale of yam cultivation and production in recent years [9]. However, many problems have also emerged during the planting process, among which the increasingly serious problem of pests and diseases of yam is particularly prominent [10]. Root rot and brown rot have a relatively large impact on the quality and yield of yam. Studies indicate that soil-borne pathogens affecting yam cultivation demonstrate progressive recurrence and intensification, resulting in yield losses ranging from 20% to 40%. Under severe infestation, losses may surpass 60% or culminate in total harvest failure [11]. Sameza et al. isolated *F.* *solani* from yam plants affected by stem rot [12]. Previous research conducted in a laboratory revealed that in the yam samples collected from Anguo City, Hebei Province, 79 pathogenic strains were isolated from the 119 samples collected. Notably, *F. solani* was the most prevalent, with 34 strains constituting 43.03% of all isolates. Therefore, *F. solani* was selected as the target microorganism in this study.

*F. solani* exhibits a broad host range, infecting crops such as cucumber, peanut, and various medicinal plants [13]. It is reported to be highly virulent compared with other Fusarium species [14]. Chemical control, including soil drenching and seed tuber treatment with fungicides such as propiconazole, difenoconazole, and dimoxystrobin, remains a common practice to manage *F. solani*-induced root rot in yam cultivation [15]. However, prolonged reliance on single-site fungicides has led to the emergence of resistant *F. solani* populations, diminishing the efficacy and sustainability of chemical interventions [16].

Biological control using microbial antagonists has emerged as an effective and environmentally sustainable alternative or supplement to chemical fungicides [17]. In recent years, microbial agents based on bacteria or fungi have been widely applied to suppress phytopathogens and mitigate disease progression [18]. For example, *B. velezensis* CX-H3 inhibits *F. graminearum* while promoting ginseng growth [19], and *B. subtilis* Pn1 shows significant biocontrol activity against Fusarium-induced root rot in *Panax notoginseng* [20]. Among biocontrol agents, *Bacillus* spp. are particularly prominent due to their multiple modes of action, including the production of extracellular enzymes (e.g., amylase, cellulase), niche competition, and synthesis of antimicrobial compounds (e.g., surfactin, iturin) that collectively inhibit pathogens such as *Macrophomina phaseolina* [21]. These mechanisms often involve hyphal deformation, suppression of spore germination, and induction of systemic resistance in host plants [22]. A comprehensive understanding of Bacillus antagonistic mechanisms is therefore essential for developing effective biocontrol strategies and assessing their ecological impact.

In this study, *B. velezensis* AP6 was screened and identified from yam rhizosphere soil. Its biocontrol potential against yam brown rot caused by *F. solani* was evaluated through in vitro antagonistic assays and field trials. Whole-genome sequencing was performed to provide preliminary insights into the molecular mechanisms underlying its antagonistic activity. These findings establish a theoretical foundation for the sustainable management of *F. solani*-associated diseases in yam using *B. velezensis*.

## 2. Materials and Methods

### 2.1. Microorganisms and Materials

The pathogens used in this study, including *F. solani*, *F. oxysporum*, *A. alternata*, *R. solani*, *Myrothecium roridum*, *Botrytis cinerea*, and *F. equiseti*, were previously isolated and preserved by the Pesticide Residue and Safety Evaluation Laboratory, College of Plant Protection, Hebei Agricultural University, Baoding, China.

All strains were routinely cultured on potato dextrose agar (PDA) and maintained in the dark at 26~28 °C. For harvesting conidia, *F. solani* colonies were gently abraded with a sterile loop under 10 mL sterile distilled water. The resulting suspension was filtered through sterile miracloth and calibrated the spore density to 1 × 10^6^ CFU/mL using a hemocytometer.

The wettable powder formulation of strain AP6 consisted of 30% sodium lignin sulfonate, 20% Tween 80, 2% calcium carbonate, 48% white carbon black, and a fermentation broth with a concentration of 2 × 10^9^ CFU/g.

### 2.2. Isolation and Screening of Biocontrol Bacterium

Rhizosphere soil samples were collected from healthy yam plants grown in a continuous cropping field in Anguo City, Hebei Province, China. The soil samples were suspended in sterile distilled water and shaken at 120 rpm for 30 min. After serial dilution to 10^−3^, 10^−4^, and 10^−5^, 100 µL aliquots of each dilution were spread onto LB agar plates and incubated at 37 °C for 2 days. Individual colonies with distinct morphologies were purified and screened for antagonistic activity against *F. solani* using the dual culture method as described previously [23]. The strain showing the strongest inhibitory effect was selected and designated strain AP6. Strain AP6 was preserved in 20% glycerol at −80 °C and has been deposited at the China General Microbiological Culture Collection Center (CGMCC) under accession number 31055.

### 2.3. In Vitro Antagonistic Activity of Strain AP6 Against Fungal Pathogens

The broad-spectrum antagonistic activity of strain AP6 against major yam pathogens was assessed using the hyphal growth inhibition assay. A mycelial plug (8 mm in diameter) of each pathogen was placed at the center of a potato dextrose agar (PDA) plate. Bacterial fermentation broth of strain AP6 was then applied at four opposing points, 2.5 cm away from the central plug. In the control group, only the pathogen was inoculated. All plates were incubated in the dark at 26~28 °C for 5~7 days. The inhibitory activity was quantified by measuring the hyphal growth inhibition rate (IR), calculated as:

$$IR=\frac{C-T}{C}\times 100\%$$

where C was the colony diameter in the control group (pathogen only), and T was the colony diameter in the treatment group [24].

### 2.4. Identification of Strain AP6

Strain AP6 was cultured on an LB agar medium to observe the colony morphology. Gram staining was performed for preliminary classification, followed by standard physiological and biochemical tests for functional identification. Furthermore, the cellular ultrastructure of strain AP6 was examined using scanning electron microscopy (SEM) [25].

### 2.5. Genome Analysis and Identification

Whole-genome sequencing of strain AP6 was conducted by Majorbio Biopharm Technology Co., Ltd. (Shanghai, China). Bioinformatics analysis was performed using data generated from the PacBio Sequel II (Pacific Biosciences of California, Inc., Menlo Park, CA, USA) and the Illumina sequencing platform (Illumina, Inc., San Diego, CA, USA). Raw sequencing data were processed and assembled to construct the complete genome using SMRT Analysis v2.3.0 and Unicycler v0.4.8. Predicted coding sequences were annotated against the GO (Blast2go), and KEGG databases (Diamond). Biosynthetic gene clusters (BGCs) were predicted with the antiSMASH software v7.0 [26].

To reconstruct the phylogenetic relationships based on the whole-genome sequence of strain AP6, single-copy orthologous genes were identified and extracted from each strain’s genome using BUSCO v5.5.0. The set of BUSCO genes that were complete, single-copy, and shared among all strains was selected for subsequent analysis. Multiple sequence alignment of the amino acid sequences for each BUSCO gene family was performed using MAFFT v7.x, and the aligned sequences were concatenated into a supermatrix in the order of the strains. A maximum-likelihood phylogenetic tree was constructed using IQ-TREE v2.2.2.7 under the best-fit nucleotide substitution model LG+F+I+R4 selected by ModelFinder. Branch support was assessed using the ultrafast bootstrap (UFBoot2) method with 1000 replicates. The resulting maximum-likelihood tree with node support values was used for subsequent phylogenetic analyses.

### 2.6. Biological Control Characteristics of Strain AP6

#### 2.6.1. Siderophore Production

Single colonies of strain AP6 cultured for 24 h were inoculated onto CAS agar plates and incubated at 37 °C for 48 h. The production of siderophores was indicated by a color shift from blue to transparent or yellow in the medium surrounding the colonies, according to the method described by Schwyn and Neilands [27].

#### 2.6.2. Secretion of Hydrolase Enzymes

Enzymatic activities of strain AP6 were evaluated as follows: amylase production was quantified following the method described by Al-Naamani et al. [28], cellulase secretion was assessed using carboxymethyl cellulose (CMC) as the substrate [29], and protease yield was determined via a proteolytic assay based on Choub et al.’s protocol [30].

#### 2.6.3. Biofilm Formation

Biofilm formation by strain AP6 was evaluated under both shaking and static cultivation conditions. After staining with crystal violet, the biofilm was solubilized in acetic acid, and its absorbance was measured at 570 nm using a UV-Vis spectrophoto-meter (Thermo Fisher Scientific, Waltham, MA, USA), with acetic acid as the blank control [31].

### 2.7. Mechanism of Action of Strain AP6 Against F. solani

#### 2.7.1. Scanning Electron Microscopy (SEM) Analysis

Fungal colonies of *F. solani* were harvested from dual-culture plates. A 5 mm × 5 mm hyphal block was carefully excised from the colony margin under sterile conditions using a scalpel. The group inoculated with *F. solani* alone was designated as the control. Fungal samples from both the treatment and control groups were then rinsed, chemically fixed, dehydrated through an ethanol gradient, dried, and sputter-coated with a thin gold layer. Hyphal morphology was subsequently examined using scanning electron microscopy (SEM) [32].

#### 2.7.2. Effects of Strain AP6 on the Germination of Spores of *F. solani*

A total of 10 μL of 1 × 10^6^ CFU/mL *F. solani* spore suspension was mixed with an equal volume of 1 × 10^9^ CFU/mL strain AP6 fermentation broth and spotted onto a sterile cellophane membrane; sterile water served as the control. Spore germination was observed under a light microscope at 0, 4, 8, 12, and 24 h after treatment, with three replicates per treatment. Furthermore, germinated spores were counted using a hemocytometer after 4 h and 24 h of incubation, and the spore germination rate was calculated accordingly [33].

### 2.8. The Fermentation Broth of Strain AP6 Induces Production of Reactive Oxygen Species (ROS) in Yam

Yam leaves were soaked in 1 × 10^9^ CFU/mL fermentation broth of strain AP6 for 24 h, removed, and rinsed with clean water. They were then soaked overnight in 0.1% nitro-blue tetrazolium chloride (NBT) buffer solution and 0.05% diaminobenzidine (DAB) buffer solution in the dark. Alcohol was used for decolorization, and blue or brown accumulation was observed and photographed. Leaves soaked in sterile water served as the control group [34].

Potted yam plants were treated by applying 10 mL of fermentation broth of strain AP6 (1 × 10^9^ CFU/mL) to the rhizosphere of each plant, with sterile water serving as the control. Leaf samples were collected at 0, 6, 12, 24, and 48 h, as well as at 5 and 7 days post-treatment. The contents of hydrogen peroxide and superoxide anion in the leaves were quantified using established plant physiology assay methods. All treatments were performed in triplicate to ensure experimental consistency and data reliability [35].

### 2.9. In Vitro Biocontrol Evaluation of Strain AP6 Against F. solani in Yam

Yam tuber segments (3 cm in length) were surface-sterilized, dried, and drilled with 1 cm deep holes. For curative efficacy, segments were first inoculated with 100 µL 1 × 10^6^ CFU/mL *F. solani* spore suspension, followed 24 h later by 100 µL 1 × 10^9^ CFU/mL fermentation broth of strain AP6. For preventive efficacy, the order was reversed: the fermentation broth of strain AP6 was applied first, followed by *F. solani* after 24 h. Sterile water served as the blank control, with three replicates per treatment. After 10 days of moist incubation at 26~28 °C, lesion areas were excised, imaged via multispectral imaging, and quantified using ImageJ software 1.54k to evaluate disease control. The formula is as follows [36]:

$$Control\;efficiency\;(\%)=\frac{Control\;group\;area-Treatment\;group\;area}{Control\;group\;area}\times 100$$

### 2.10. Effect of Strain AP6 on F. solani Spores in Soil

Soil collected from the experimental field was sieved and dried. After inoculating it with 2 mL of a mixed suspension containing 1 × 10^6^ CFU/mL of *F. solani*, 2 mL of 1 × 10^7^ CFU/mL fermentation broth of strain AP6 was uniformly inoculated. Sterile water and LB medium treatments were used as controls. An additional 2 mL of the 1 × 10^7^ CFU/mL fermentation broth of strain AP6 was added every 20 days for a total of five applications. Ten days after each application, a 1.00 g soil sample was taken for concentration gradient dilution and plating in order to observe the number of spores present in the soil [37].

### 2.11. Field Experiment Design and Evaluation Methods

#### 2.11.1. Field Experiment Design

The field experiment was conducted at the yam planting base in Dananliu Village, Anguo City, Hebei Province (38°42′48″ N, 115°33′30″ E), encompassing land preparation and sowing. In 2023, 3 processing methods were designed, and in 2024, 6 processing methods were designed (Table 1), with a blank control group serving as the reference. The emergence rate of yam seedlings was recorded throughout the trial. Drip irrigation was implemented every 20 days starting from 19 July 2023, for a total of 2 applications. Drip irrigation was implemented every 20 days starting from 29 June 2024, for a total of 3 applications. Routine field management practices were maintained consistently throughout the experiment. After reaching maturity, yams were harvested to assess yield and disease-related parameters.

The effective viable bacteria count of the fermentation broth of strain AP6 is ≥2 × 10^9^ CFU/mL. Dilute it 300 times and apply it by drip irrigation at a rate of 2 L/667 m^2^; the microbial agent Vigorous, sold on the market, has a viable bacteria count of ≥5 × 10^9^ CFU/mL. Dilute it 500 times and apply it by drip irrigation at a rate of 1 L/667 m^2^. Soak seeds in it for 30 min, dry them, and then sow them; the active ingredients and content of the chemical agent Prochloraz are 450 g/L. Apply it by drip irrigation at a rate of 30 g/667 m^2^. The effective viable bacterial count of the wettable powder formulation of AP6 is ≥7 × 10^9^ CFU/g. For application, administer the product via drip irrigation at a rate of 330 g/667 m^2^. To prepare the seeds, soak them in a solution at a concentration of 1 g/L for 30 min, then allow them to dry before sowing. For the metalaxyl-M and fludioxonil, the active ingredient content is 62.5 g/L. Apply this product through drip irrigation at a rate of 200 g/667 m^2^. Similarly, soak the seeds in a solution at a concentration of 1 g/L for 30 min, dry them, and then proceed with sowing [38].

#### 2.11.2. Yield Measurement

At harvest, the yield was assessed by collecting and weighing all yams from two consecutive 10-m rows per plot. From these rows, 10 yams were randomly sampled per treatment, with three replicates. Disease severity was scored based on the established grading standard for yam brown rot (Table 2). The disease index and control efficiency were then calculated using the following formulas [39]:

$$Rate\;of\;growth\;\left(\%\right)=\frac{Treatment\;group-Control\;group}{Control\;group}\times 100$$

$$Disease\;index=\frac{\sum (Severity\;grade\times Number\;of\;plants\;at\;corresponding\;grade)}{Highest\;incidence\;grade\times Total\;number\;of\;investigated\;plants}\times 100$$

$$Control\;efficiency\;\left(\%\right)=\frac{Control\;group-Treatment\;group}{Control\;group}\times 100$$

### 2.12. Statistical Analysis

Statistical analysis was performed using SPSS version 25.0. Comparisons between two groups were conducted using independent samples *t*-tests to evaluate differences in means between experimental and control groups, with statistical significance set at *p* < 0.05. For multiple group comparisons, one-way analysis of variance (ANOVA) followed by the least significant difference (LSD) test was employed, maintaining the same significance threshold (*p* < 0.05) for all pairwise comparisons.

## 3. Results

### 3.1. Screening of Biocontrol Strains Against F. solani

**Figure 1:**
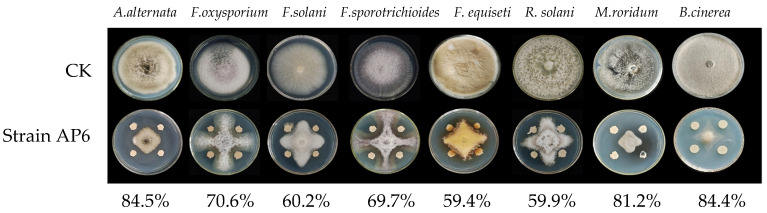
Inhibition effect of strain AP6 on a variety of pathogens.

The mycelial growth rate method was employed to assess the antagonistic activity of the isolated biocontrol bacteria against *F. solani*. Among the 71 isolated strains, strain AP6 exhibited the highest inhibitory activity against *F. solani*, with an inhibition rate of 60.2%. Consequently, strain AP6 was selected as the target strain for subsequent investigations (Figure 1).

### 3.2. In Vitro Broad-Spectrum Antagonistic Activity of Strain AP6

Broad-spectrum antagonistic assays revealed that strain AP6 exhibited inhibition rates of 59.4% to 84.5% against tested major yam pathogens, with the most potent effect (84.5%) observed against *A. alternata*. These results demonstrate its strong, broad- spectrum efficacy (Figure 1).

### 3.3. Identification of Strain AP6

**Figure 2:**
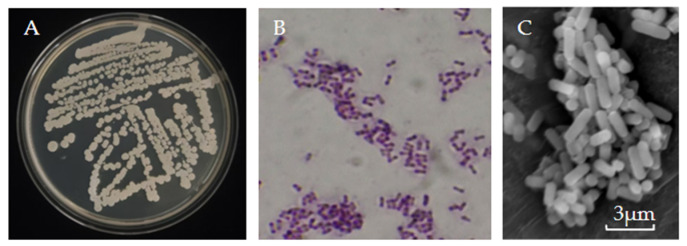
Colony morphology and micro-morphology of strain AP6. (**A**) Colonial morphology of strain AP6 on LB agar after 48 h incubation at 28 °C. (**B**) Gram staining of strain AP6. (**C**) Bacterial morphology of strain AP6 by SEM examination (bar = 3 µm).

As shown in Figure 2A,B, colonies of strain AP6 grown on LB agar were circular to oval, with a smooth, milky white, and opaque surface. The central region of the colonies appeared wrinkled and viscous, accompanied by a characteristic odor. Gram staining revealed purple cells, confirming that strain AP6 is a Gram-positive bacterium. Scanning electron microscopy (SEM) further showed that the cells were rod-shaped (Figure 2C).

Physiological and biochemical characterization showed that strain AP6 did not produce acid from glucose, maltose, sucrose, or xylose. Tests for catalase activity, citrate utilization, and hydrolysis of arginine, ornithine, and lysine were all negative, and no hydrogen sulfide production was detected. However, strain AP6 was capable of hydrolyzing aesculin, urea, and starch, fermenting lactose, and producing a positive Voges–Proskauer (VP) reaction (Table 3). These characteristics are consistent with the description of the genus Bacillus as outlined in the Manual for Systematic Identification of Common Bacteria.

### 3.4. Whole-Genome Analysis and Identification of Strain AP6

**Figure 3:**
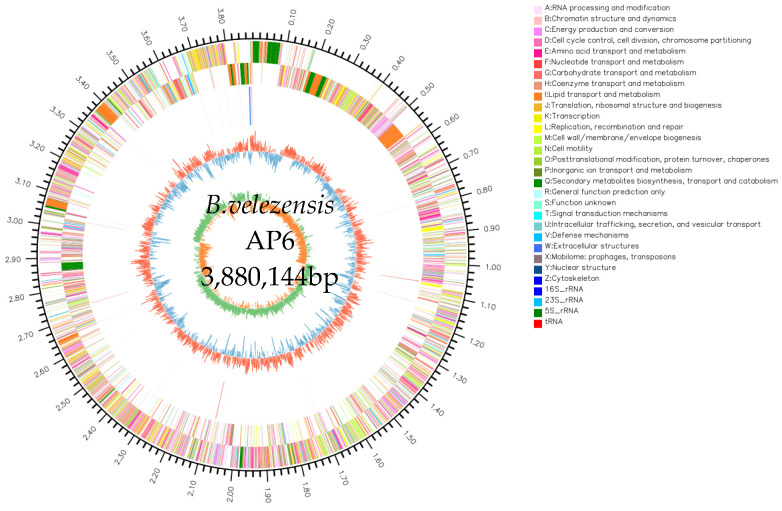
Genome map of strain AP6. The circles from 1 to 6 (outer to inner) represent markers of genome size, CDS on the forward strand, CDS on the reverse strand, rRNA and tRNA, GC content, and GC-skew.

To elucidate the genomic characteristics and predict the potential biocontrol mechanisms of strain AP6, its whole genome was sequenced using a combination of Illumina sequencing and the PacBio Sequel II platform. A circular genome map was generated with Circos software v0.69-9. The complete genome of strain AP6 is 3,880,144 bp in length, with an average GC content of 46.44%. It contains 3675 coding sequences (CDS), 79 tRNA genes, and 9 rRNA genes (Figure 3).

**Figure 4:**
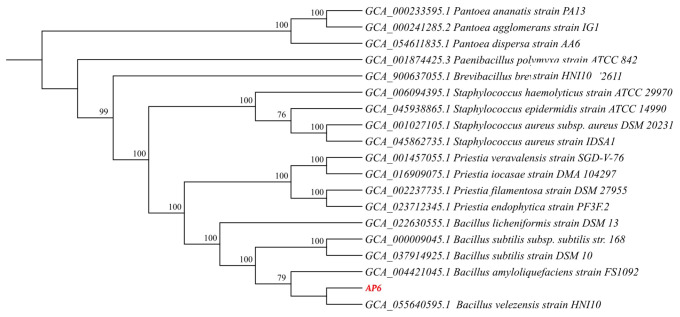
Phylogenetic analysis of strain AP6. A phylogenetic tree based on whole-genome sequences.

The whole-genome sequence of strain AP6 has been deposited in the GenBank database under accession number JBVYPZ000000000. Phylogenetic analysis based on whole-genome sequences of strain AP6 and related species placed strain AP6 in a clade closely related to *Bacillus velezensis* HNI10 (Figure 4). The results of ANI (Average Nucleotide Identity) analysis and dDDH (Digital DNA-DNA Hybridization) analysis indicated that strain AP6 is *Bacillus velezensis* (Supplementary Tables S1 and S2).

**Figure 5:**
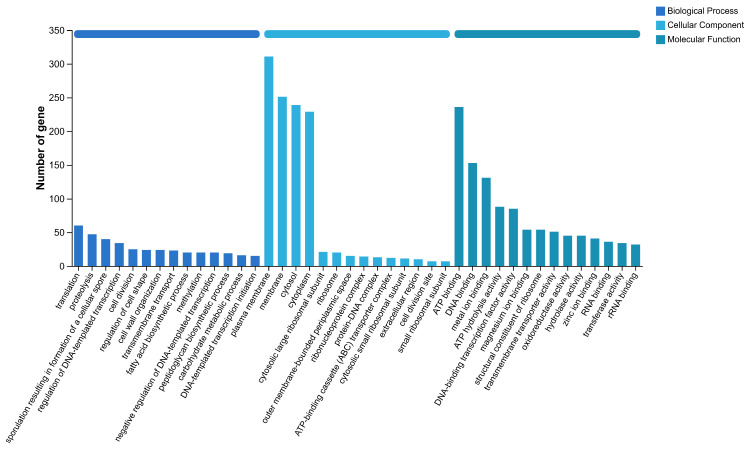
The GO classification of strain AP6 genome.

**Figure 6:**
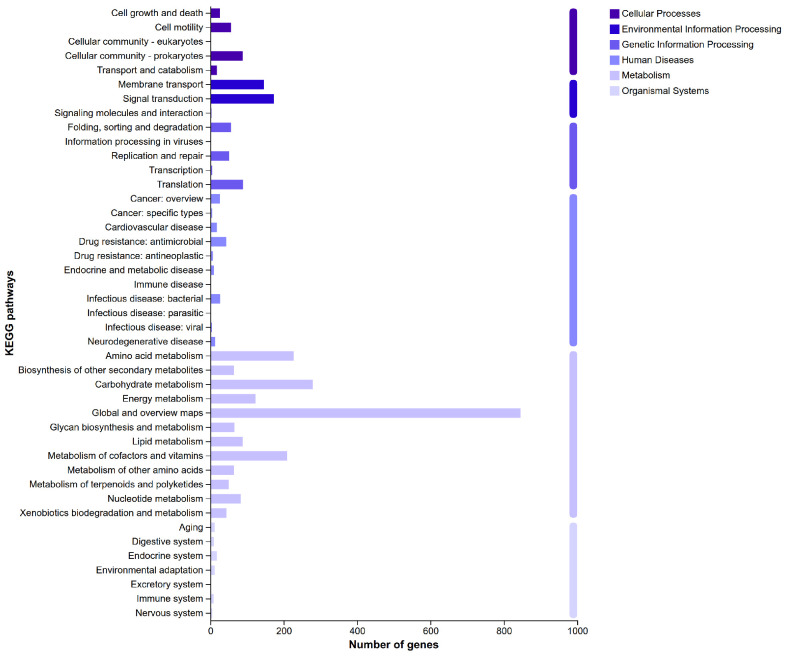
The KEGG annotation of strain AP6 genome.

The proportion of total coding genes annotated in the GO database is categorized into biological processes, cellular components, and molecular functions, as shown in Figure 5. Additionally, KEGG annotation identified 2826 genes, with the top three categories being global and overview maps (845 genes), carbohydrate metabolism (279 genes), and amino acid metabolism (227 genes) (Figure 6).

**Figure 7:**
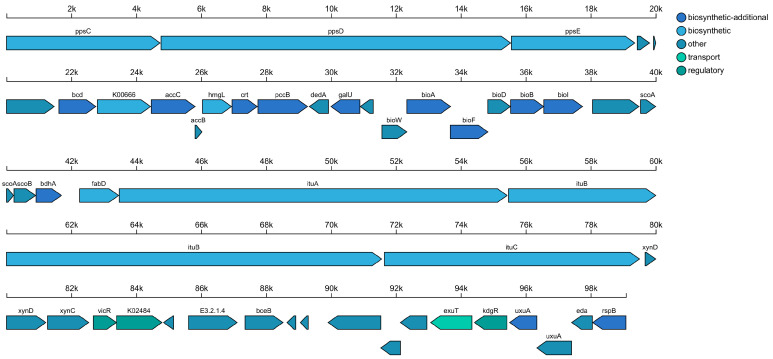
Linear map of gene clusters for secondary metabolite synthesis. The graph shows all the genes in the predicted gene cluster. The colors of the different annotated genes represent the functional classification of the genes within this secondary metabolic product synthesis gene cluster.

A total of 18 biosynthetic gene clusters (BGCs) were predicted in the strain AP6 genome using antiSMASH software v7.0 (Figure 7 and Table 4). These BGCs are classified into eight groups: four trans-AT PKS and trans-AT PKS-like clusters, three nonribosomal peptide synthetases (NRPS), two terpenes, as well as PKS-like, RiPP-like, T3PKS, T1PKS, and other types. Notably, five BGCs exhibited high similarity to known clusters responsible for producing macrolactin H (100%), bacillaene (100%), butirosin A/butirosin B (100%), and fengycin (86%). Some BGCs displayed very low similarity to known clusters or were uncharacterized, suggesting that strain AP6 may synthesize novel metabolites.

### 3.5. Biocontrol-Related Traits of Strain AP6

**Figure 8:**
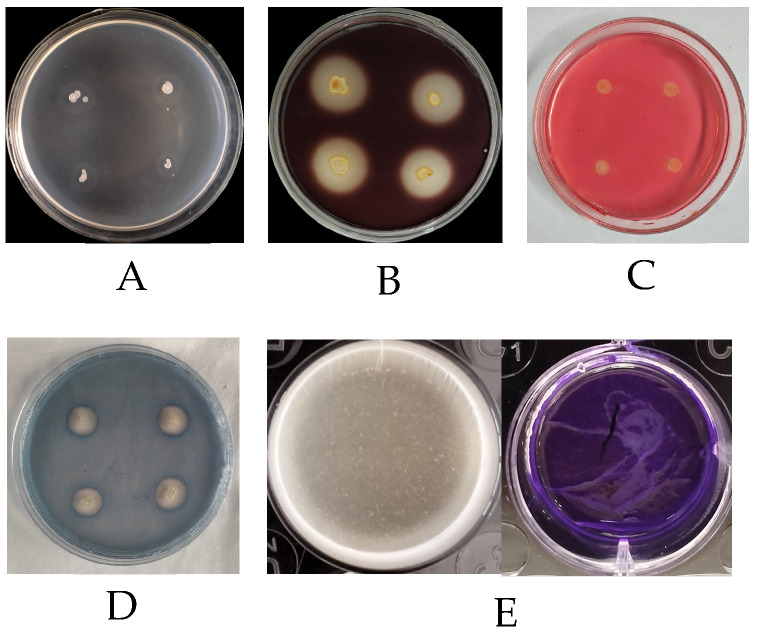
Growth-promoting substances secreted by strain AP6. (**A**) amylase, (**B**) protease, (**C**) cellulase, (**D**) siderophile, (**E**) biofilm formed.

Strain AP6 can secrete extracellular hydrolases, including amylase, cellulase, and protease, produce siderophores, and form biofilms. These traits indicate that strain AP6 can suppress diseases indirectly (Figure 8).

### 3.6. Mechanism of Action of Strain AP6 Against F. solani

#### 3.6.1. Effect of Strain AP6 on the Mycelial Morphology of *F. solani*

**Figure 9:**
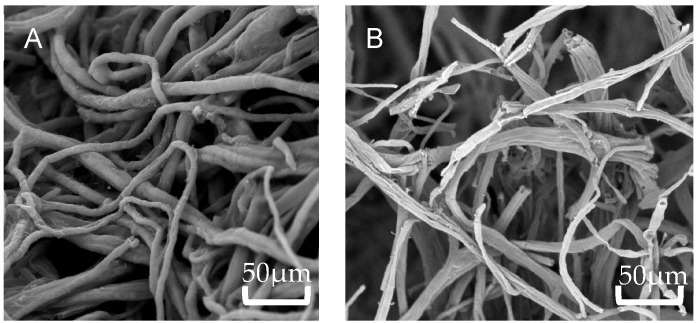
SEM of hyphal morphology of *F. solani* under strain AP6 stress. (**A**) control group, (**B**) treatment group.

The mycelial structure at the colony edges was examined using SEM. Observation indicated that while the control mycelia remained intact and smooth, those of strain AP6 treated *F. solani* displayed severe deformities such as twisting, folding, collapse, and heightened branching (Figure 9).

#### 3.6.2. Effect of Strain AP6 on Spore Germination of *F. solani*

**Figure 10:**
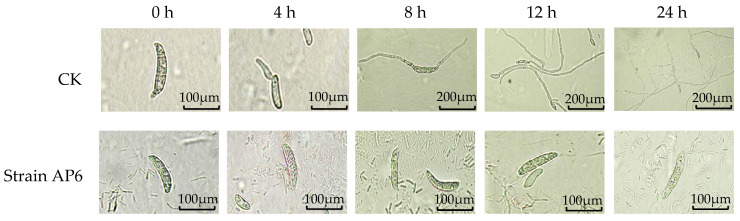
Effects of strains AP6 on the spore germination of *F. solani*.

After 24 h of treatment with strain AP6, spores remained completely ungerminated. In contrast, spores in the control group progressed through distinct developmental stages: unilateral germ tube emergence was observed at 4 h, followed by bilateral germination and initial hyphal growth at 8 h, hyphal elongation by 12 h, and the formation of fully developed, conidia-producing hyphae at 24 h (Figure 10). Statistical analysis revealed that the inhibition of spore germination by strain AP6 began as early as 4 h post-treatment, with the germination rate suppressed to only 6.6% at 24 h post-treatment (Table 5).

### 3.7. Effect of AP6 Fermentation Broth on Reactive Oxygen Species (ROS) Production in Yam

**Figure 11:**
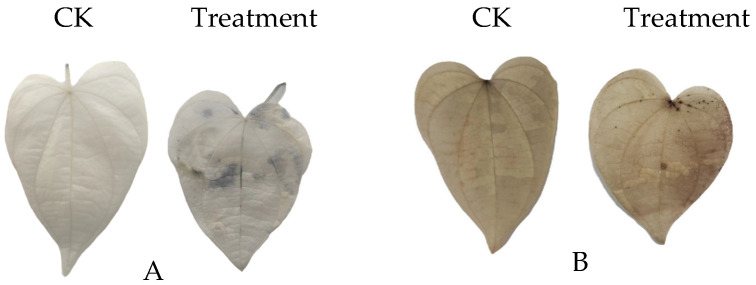
Strain AP6 induced ROS production in yam. (**A**) NBT, (**B**) DAB.

**Figure 12:**
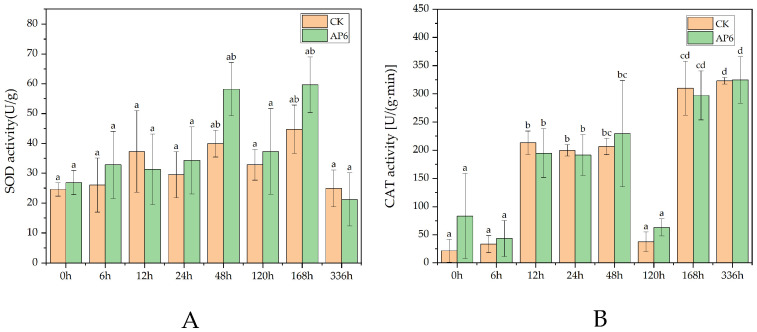
Strain AP6 induced ROS production in yam. (**A**) SOD, (**B**) CAT. The data in the table are the mean ± standard deviation; different lowercase letters indicate the level of significance of the difference (*p* < 0.05).

The impact of strain AP6 on ROS levels in yam was analyzed using histochemical staining with DAB and NBT. Following treatment with the fermentation broth of strain AP6, yam leaves developed distinct brown and blue precipitates, corresponding to the accumulation of hydrogen peroxide and superoxide anion, respectively. No precipitate formation was observed in untreated control leaves (Figure 11). Similarly, in yam leaves treated with fermentation broth of strain AP6, catalase (CAT) content peaked on the 14th day, while superoxide dismutase (SOD) content reached its maximum on the 2nd and 7th days (Figure 12).

### 3.8. In Vitro Effect of Biocontrol Evaluation of Strain AP6 Against F. solani in Yam

**Figure 13:**
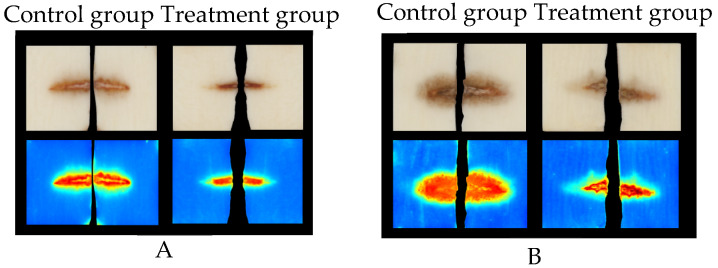
Preventive and curative efficacy of strain AP6 against *F. solani* in yam. (**A**) preventive efficacy, (**B**) curative efficacy. In the figure, the red and yellow parts represent the portions of yam diseased after inoculation with *F. solani*, and the blue part represents the portion not infected.

In vitro assays demonstrated that strain AP6 exhibited both curative and preventive efficacy against *F. solani*, with control efficiency of 67.6% and 77.3%, respectively, indicating its strong protective and notable curative activities (Figure 13 and Table 6).

### 3.9. Effect of Strain AP6 on F. solani Spores in Soil

**Figure 14:**
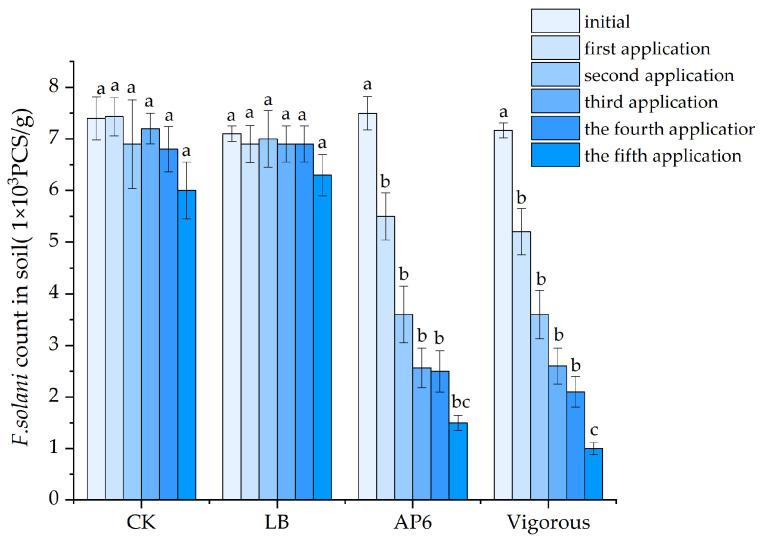
Strain AP6 on *F. solani* spores in soil. The data in the table are the mean ± standard deviation; different lowercase letters indicate the level of significance of the difference (*p* < 0.05).

As shown in Figure 14, by comparing the number of spores in the soil after the fifth application of the pesticide, it was observed that Vigorous exhibited the best inhibitory effect on the spores of the *F. solani*. Compared with the control, the number of spores for strain AP6 decreased significantly and continued to decline with each subsequent application of strain AP6. After five applications, the number of spores decreased from an initial count of 7.0 × 10^3^ spores per gram to 1.5 × 10^3^ spores per gram.

### 3.10. Field Control Efficacy of Wettable Powder of Strain AP6 Against Yam Brown Rot and Root Rot Under Different Application Methods

#### 3.10.1. Effect of Different Agent Treatments on Yam Yield

Yams were harvested on 19 November 2023, and yields were recorded. As shown in Table 7, the highest yields were obtained from treatments involving fermentation broth of strain AP6 drip irrigation, reaching 2522 kg per 667 m^2^ (T1), respectively. These represented yield increase rates of 14.9% over the control. Vigorous drip irrigation (T2) and Prochloraz (T3) broth drip irrigation, reaching 2493 kg and 2210 kg per 667 m^2^. These represented yield increase rates of 13.6% and 0.7% over the control.

Yams were harvested on 16 November 2024, and the yields were recorded. As shown in Table 8, the highest yields were recorded for treatments involving wettable powder of strain AP6 seed soaking supplemented with drip irrigation of metalaxyl-M and fludioxonil, reaching 2070 kg and 2119 kg per 667 m^2^ (T3 and T4), respectively. These represent yield increases of 7.6% and 5.3% over the control. In contrast, the other chemical treatments tested did not exhibit significant yield-promoting effects.

#### 3.10.2. Effect of Different Agent Treatments on Yam Brown Rot

Field trial results show that all pesticide application methods provided some control of yam brown rot (Table 9). The most effective strategy was treatment T2 (Fermentation broth of strain AP6 drip irrigation), with a control rate of 62.9%; it was statistically equivalent to treatment T1 (Vigorous drip irrigation), and both treatments were significantly superior to the control.

Field trial results show that all pesticide application methods provided some control of yam brown rot (Table 10). The most effective strategy was treatment T2 (Wettable powder of strain AP6 seed soaking combined with metalaxyl-M and fludioxonil drip irrigation), with a control rate of 79.3%; it was statistically equivalent to treatment T1 (Metalaxyl-M and fludioxonil seed soaked with wettable powder of strain AP6 irrigation), and both were significantly superior to the control.

## 4. Discussion

The recent increase in yam demand has led to a corresponding expansion in cultivation area. This trend has been paralleled by a rise in both the incidence and severity of yam diseases. The use of chemical pesticides to combat these diseases, however, raises serious concerns about product quality and pesticide residues [40]. Therefore, the use of biological control for disease management presents a viable alternative. In this study, *B. velezensis* was isolated from the rhizosphere soil of yam, and its ability to control yam brown rot was evaluated. Based on the cultural characteristics and whole-genome sequencing of strain AP6, the results identify it as *B. velezensis*. Numerous reports have highlighted the biological control capabilities of Bacillus strains against various plant diseases. For instance, the biocontrol agent *B. velezensis* L11-7 has been demonstrated to control passion fruit stem rot while also promoting plant growth [25]. Likewise, *B. subtilis* C3 has shown efficacy in controlling kiwifruit root rot and restoring rhizosphere ecological functions [41]. However, there are relatively few studies on biocontrol bacteria for yam. Strain AP6 demonstrated significant control efficacy against yam brown rot, confirming its potential as a biocontrol resource. Notably, this finding aligns with current global research priorities in sustainable plant protection. To our knowledge, this study provides the first report of a Bacillus strain exhibiting control activity against yam brown rot.

The use of biocontrol bacteria to control plant diseases is receiving increasing attention [42]. Chinese herbal medicines serve as a rich source of diverse bioactive compounds, which have been widely utilized. Therefore, research on biocontrol bacteria in Chinese herbal medicinal plants is also expanding. *B. velezensis* promotes plant growth through multiple mechanisms, including enhanced stress tolerance and direct inhibition of phytopathogens [43]. This study verified that strain AP6 can produce hydrolases, such as proteases, amylases, and cellulases, as well as siderophores, and can form biofilms. Hydrolases contribute to the degradation of fungal cell walls, thereby inhibiting pathogen growth [44]. Furthermore, siderophores chelate ferric iron in the environment, which not only inhibits pathogen growth but also aids plants in acquiring essential trace elements [45]. The formation of biofilms by biocontrol bacteria facilitates their colonization on plants, enabling them to occupy ecological niches and inhibit pathogen proliferation [31].

Applying beneficial microorganisms with antagonistic activity can effectively protect host plants from infectious pathogens [30]. When studying the mechanism of action of strain AP6 against *F. solani* in yam, it was found that strain AP6 can inhibit spore germination and mycelial growth, thereby preventing infection. The conidia of *F. solani* can remain viable in the soil for several years, making the inhibition of spore germination crucial for effective disease control [46]. Additionally, the application of strain AP6 led to increased levels of hydrogen peroxide and superoxide anion radicals in yam, indicating an activated plant stress response. Future studies will employ techniques such as q-PCR to investigate whether treatment with strain AP6 activates specific defense pathways, such as the salicylic acid (SA) signaling pathway, in yam plants. Moreover, the combined use of the fermentation broth of strain AP6 and the wettable powder formulation of strain AP6 with metalaxyl-M and fludioxonil improved both the yield and control efficacy against brown rot. Studies have shown that in field trials, the wettable powder formulation of *B. velezensis* strain F0b exhibited significant control efficacy against *B. cinerea*, with control effects ranging from 50.58% to 73.14% [38]. Although strain AP6 demonstrates promising control efficacy against yam brown rot, two years of field trials remain limited. Therefore, we plan to conduct further field studies to validate the stability and broader adaptability of its control performance.

Genome analysis of strain AP6 revealed the presence of biosynthetic gene clusters (BGCs) associated with the production of antibiotics, such as fengycin, surfactin, and bacillibactin. These BGCs exhibited 100% similarity with known genes that demonstrate antagonistic activity [47]. Importantly, the presence of these unknown compounds suggests that strain AP6 may produce novel antimicrobial agents. The mechanisms of action for these compounds warrant further investigation, potentially through gene knockout techniques [48].

## 5. Conclusions

This study identifies *B. velezensis* AP6, isolated from yam rhizosphere soil, as a promising biocontrol agent against yam brown rot caused by *F. solani*. Its efficacy stems from a dual mechanism: direct inhibition of pathogen growth (via hyphal deformation and suppression of spore germination) and indirect protection through induced systemic resistance in the host plant. The significant disease control achieved by combining strain AP6 with reduced rates of metalaxyl-M and fludioxonil in field trials presents a viable strategy for sustainable disease management. These findings not only highlight the potential of strain AP6-based formulations but also lay the groundwork for future investigations aimed at characterizing the specific antimicrobial compounds and molecular pathways responsible for its antagonistic activity.

## Figures and Tables

**Table 1:** Daejeon trial design.

Treatment Number	2023 Year	2024 Year
T1	Fermentation broth of strain AP6 drip irrigation	Metalaxyl-M and fludioxonil seed soaked, Wettable powder of strain AP6 drip irrigation
T2	Vigorous drip irrigation	Wettable powder of strain AP6 seed soaked, Metalaxyl-M and fludioxonil drip irrigation
T3	Prochloraz drip irrigation	Metalaxyl-M and fludioxonil seed soaked, Wettable powder of strain AP6 + metalaxyl-M and fludioxonil drip irrigation
T4	Blank control	Wettable powder of strain AP6 + metalaxyl-M and fludioxonil seed soaked, Metalaxyl-M and fludioxonil drip irrigation
T5		Metalaxyl-M and fludioxonil seed soaked, Metalaxyl-M and fludioxonil drip irrigation
T6		Blank control

Note: “+” stands for combined use.

**Table 2:** Disease grading criteria.

Level	Brown Rot Disease Type
0	No diseased spots on tubers
1	There were a few spots on the tubers, less than 10% of the area
3	There are more diseased spots on tubers, and the area of diseased spots accounts for 10–25% of the total area of tubers
5	There were many and large lesions on tubers, and the area of lesions accounted for 25–50% of the total area of tubers
7	The tuberous spots are contiguous, and the area of lesions accounts for 50–70% of the total area of the tuber
9	The area of disease spots on tubers accounted for more than 70% of the total area of tubers

**Table 3:** Physiological and biochemical identification results of strain AP6.

Physiological and Biochemical Index	Reaction Characteristics
glucose	−
maltose sugar	−
sucrose	−
xylose	−
Oxidative contact enzyme	−
aesculin	+
Hydrolysis of starch	+
V.P	+
Hydrogen sulfide	−
ONPG	+
urea	+
Simon’s citrate	−
arginine	−
ornithine	−
lysine	−

Note: “+” indicates positive; “−” indicates negative.

**Table 4:** Secondary metabolite synthesis gene clusters of *B. velezensis* AP6.

Cluster ID	Type	Start	End	Similar Metabolite	Similarity (%)	Gene No
Cluster 1	NRPS	2	99,082	fengycin	86	47
Cluster 2	transAT-PKS	171,031	271,297	bacillaene	100	43
Cluster 3	transAT-PKS	490,461	578,695	macrolactin H	100	44
Cluster 4	terpene	857,632	878,373	-	-	23
Cluster 5	PKS-like	961,152	1,002,397	butirosin A/butirosin B	7	41
Cluster 6	other	314,161	355,580	bacilysin	100	42
Cluster 7	RiPP-like	880,134	931,925	bacillibactin	100	45
Cluster 8	transAT-PKS	541,773	576,849	difficidin	40	29
Cluster 9	NRPS	185,708	251,116	surfactin	78	40
Cluster 10	transAT-PKS-like	1	45,900	difficidin	53	30
Cluster 11	T3PKS	161,505	202,606	-	-	50
Cluster 12	terpene	253,027	274,911	-	-	22
Cluster 13	transAT-PKS	1	52,967	difficidin	20	28
Cluster 14	T1PKS	1	30,125	macrobrevin	20	21
Cluster 15	ransAT-PKS-like	1	23,405	difficidin	26	4
Cluster 16	NRPS	1	13,788	fengycin	26	3
Cluster 17	transAT-PKS-like	1	8335	elansolid A1	10	2
Cluster 18	transAT-PKS-like	2	5264	-	-	2

**Table 5:** Effect of strain AP6 on spore germination of *F. solani*.

Strain Number	4 h	24 h
Strain AP6	0 ± 0.0 a	7.6 ± 6.6 a
CK	61.2 ± 1.4 b	90.0 ± 7.7 c

Note: The data in the table are the mean ± standard deviation; different lowercase letters indicate the level of significance of the difference (*p* < 0.05).

**Table 6:** Mode of action of strain AP6 against *F. solani*.

Mode of Action	Strain Number	Lesion Area (mm^2^)	Control Effect (%)
Curative efficacy	Strain AP6	31.4 ± 3.8 a	53.0 ± 5.7 a
	CK	66.7 ± 1.1 c	-
Preventive efficacy	Strain AP6	15.8 ± 0.8 a	65.6 ± 1.8 a
	CK	46.0 ± 4.8 c	-

Note: The data in the table are the mean ± standard deviation; different lowercase letters indicate the level of significance of the difference (*p* < 0.05).

**Table 7:** Effects of different chemical treatments on the yield of yam (2023).

Treatment Number	Treatment Name	Yield (kg/667 m^2^)	Yield Increase (%)
T1	Fermentation broth drip irrigation of strain	2522 ± 6	14.9 ± 2.6
T2	Vigorous drip irrigation	2493 ± 7	13.6 ± 2.8
T3	Prochloraz drip irrigation	2210 ± 5	0.7 ± 2.1
T4	Blank control	2195 ± 8	-

Note: The data in the table are the mean ± standard deviation.

**Table 8:** Effects of different chemical treatments on the yield of yam (2024).

Treatment Number	Treatment Name	Yield (kg/667 m^2^)	Yield Increase (%)
T1	Metalaxyl-M and fludioxonil seed soaked Wettable powder of strain AP6 irrigation	2015 ± 2 c	0.2 ± 0.1 c
T2	Wettable powder of strain AP6 seed soaked Metalaxyl-M and fludioxonil drip irrigation	2010 ± 3 c	-
T3	Metalaxyl-M and fludioxonil seed soaked, Wettable powder of strain AP6 + metalaxyl-M and fludioxonil drip irrigation	2070 ± 10 b	2.9 ± 0.5 b
T4	Wettable powder of strain AP6 + metalaxyl-M and fludioxonil seed soaked, Metalaxyl-M and fludioxonil drip irrigation	2119 ± 7 a	5.3 ± 2.6 a
T5	Metalaxyl-M and fludioxonil seed soaked, Metalaxyl-M and fludioxonil drip irrigation	1885 ± 2 d	-
T6	Blank control	2012 ± 15 c	-

Note:”+” stands for combined use, “-” indicates no increase in production. The data in the table are the mean ± standard deviation; different lowercase letters indicate the level of significance of the difference (*p* < 0.05).

**Table 9:** Effects of different chemical treatments on brown rot of yam (2023).

Treatment Number	Disease Index	Control Effect (%)
T1	5.56 ± 1.92 a	57.1 ± 14.9 a
T2	4.81 ± 0.64 a	62.9 ± 5.0 a
T3	8.52 ± 1.70 b	34.3 ± 13.1 b
T4	12.96 ± 1.70 c	-

Note: The data in the table are the mean ± standard deviation; different lowercase letters indicate the level of significance of the difference (*p* < 0.05).

**Table 10:** Effects of different chemical treatments on brown rot of yam (2024).

Treatment Number	Disease Index	Control Effect (%)
T1	4.07 ± 1.28 bc	67.7 ± 10.2 abc
T2	1.85 ± 0.64 a	79.3 ± 5.1 a
T3	3.33 ± 1.11 ab	73.5 ± 8.9 ab
T4	9.63 ± 2.31 de	23.5 ± 10.2 e
T5	8.19 ± 0.71 d	35.0 ± 5.6 de
T6	12.59 ± 1.70 f	-

Note: The data in the table are the mean ± standard deviation; different lowercase letters indicate the level of significance of the difference (*p* < 0.05).

## Data Availability

The original contributions presented in this study are included in the article. Further inquiries can be directed to the corresponding authors.

## Supplementary Materials

The following supporting information can be downloaded at: https://www.mdpi.com/article/10.3390/jof12050345/s1, Table S1: ANIb similarity analysis results of strain AP6; Table S2: dDDH analysis results of strain AP6.
