# Supplementary material for: Biocontrol Efficacy and Mechanisms of Bacillus velezensis AP6 Against Brown Rot in Yam by Fusarium solani
Source: J Fungi (Basel). 2026 May 7;12(5):345. doi: 10.3390/jof12050345 (PMC13208842; doi:10.3390/jof12050345)
Supplement: Supplementary file 1 [file jof-12-00345-s001.zip › jof-4285843-supplementary.pdf]

Table S1. ANIb similarity analysis results of strain AP6.

Strain	AP6	*B. Velezensis* L-H15	*B. Velezensis* NKYL29	*B. velezensis* G341	*B. Velezensis* B26
AP6	-	94.89	98.16	98.07	98.01
B. velezensis L-H15	-	-	97.55	97.61	97.64
B. Velezensis NKYL29	-	-	-	98.20	98.24
B. velezensis G341	-	-	-	-	99.17
B. velezensis B26	-	-	-	-	-

Note: The values in the table are the ANIb (Average Nucleotide Identity based on BLAST) similarity (%) between strains and between strains and the fasta file; "-" indicates self-alignment, which has no practical analytical significance; ANIb similarity ≥ 96% can be determined as the same species.

Table S2. dDDH analysis results of strain AP6.

Subject	d_4	C.I. d_4	Diff. G+C Percent
GCA_001685645.1	86.3	[83.6-88.5]	0.84
GCF_001461845.1	82.8	[80.0-85.3]	1.03
CP072310.1	77.4	[74.4-80.1]	0.72
JAZIAW000000000	77.3	[74.3-80.0]	0.71

Note: The values in the table are pairwise dDDH values (%) between strains, along with their confidence intervals (C.I.) for three different GBDP formulas; \(d_4\) (GGDC formula 2): sum of all identities found in HSPs divided by overall HSP length (independent of genome length, robust against incomplete draft genomes); dDDH value ≥ 70% can be determined as the same species.
